# Prescriptions and Dispensing

**Published:** 1908-07-04

**Authors:** 


### THERAPEUTICS.

### PRESCRIPTIONS AND DISPENSING.

### Pills (*continued*).

If, as sometimes happens, Blaud's pill is prescribed in combination with other ingredients, the latter must be added at an appropriate stage in the making. A few instances may be given:—

| | |
|---|---|
| Acidi Arseniosi ... ... ... ... | gr. $\frac{1}{50}$ |
| Strychninæ ... ... ... ... | gr. $\frac{1}{50}$ |
| Aloin ... ... ... ... ... | gr. $\frac{1}{10}$ |
| Pilulæ Ferri ... ... ... ... | gr. iv. |

Fiat pilula j. Mitte xxxvj.

The three first ingredients should be thoroughly mixed together, and then with the mixed syrup, glycerin, and water. They will not interfere at all with the subsequent reaction when the sodium carbonate and ferrous sulphate powders are added, and their proper distribution is effectually secured.

| | |
|---|---|
| Extracti Aloes Barbadensis ... ... | gr. xij. |
| Pulveris Nucis Vomicæ ... ... | gr. vj. |
| Pilulæ Ferri ... ... ... ad | ʒiv. |

Misce. Fiant pilulæ xlviij.

The nux vomica powder may be mixed with the liquids as in the previous case; but as the extract of aloes would appreciably retard the reaction between the sodium carbonate and the ferrous sulphate it should be mixed with the acacia and tragacanth, and added with them when the change is complete.

| | |
|---|---|
| Manganesi Dioxidi precipitati ... | gr. ij. |
| Pilulæ Ferri ... ... ... ... | gr. iij. |

Fiat pilula j. Tales xxiv.

In this case the iron pill-mass should be made first, and the manganese dioxide added afterwards, as otherwise reaction would almost certainly occur to some extent between it and the sodium carbonate, leaving some of the ferrous sulphate unconverted into carbonate of iron. It is not necessary to multiply examples of prescriptions for pills; ingenuity may be required in making many of them up satisfactorily; the two essentials for good pill-making are common-sense and practice. There are various small matters in practical work in which the difference between a good and a bad pill-maker are likely to be shown.

### Pill-machines.

The pill-machines likely to be used in general practice are made to cut twenty-four pills at one time. If a larger number than this is to be made the mass should always be divided into the requisite number of parts by *weighing*, the eye alone never being trusted for their equal size. If 5-grain pills are to be made, and the machine to be used is of 5-grain size, it is only necessary to roll out the mass into a cylinder of the exact length which corresponds to the number of pills that are to be made, and cut them with a rapid to-and-fro movement of the cutter, using gentle pressure at first and gradually increasing it. After a little practice the pills so produced will require no further rounding. The production of a cylinder of perfectly uniform thickness, however, requires some care; application of pressure at the ends only of the roller tends to make the ends of the cylinder of pill-mass rather thinner than the middle; and from the fact that the middle portions of the roller and of the bed of the pill-machine get more wear than the outer portions, they gradually come to be slightly hollowed; this also makes the middle part of the cylinder thicker than the ends.

It is usually necessary, therefore, when the mass is rolled out to rather less than the required length, to apply the roller in a direction at right angles to the usual one; that is to say, with one end towards, and the other directly away from, the operator, so that pressure is only put on to the cylinder over a portion equal in length to the width of the pill-roller. Any irregularities in thickness can thus be corrected, and the cylinder brought to perfect uniformity as judged by the eye.

It often happens that pills have to be made on a machine that is intended for a larger pill, say 3-grain pills on a 4-grain or 5-grain machine. In this case the cutting will not as a rule give round pills, but elongated ones. It is then usually necessary to make each pill approximately round by pressure between finger and thumb, in order that they may run when rotated under the rounder or finisher.

The knack of using the rounder is not difficult to acquire; a circular rotatory motion is required, and the pressure should be very gentle at first; then gradually increased to a maximum which varies with the hardness of the mass; and again diminished as the motion is reduced and stopped. In rolling and rounding pills, as well as in making the mass, the heat generated by friction plays an important part; a very hard mass may be made quite plastic by vigorous handling, and a few minutes after the pills are finished they will be quite hard again, and in no danger of losing their shape.

It is usually desirable to use a little powder to prevent the mass sticking to the machine in rolling and cutting, and to prevent the pills sticking together in rounding. Various powders are used for the purpose; probably the best is a mixture of equal proportions of starch and French chalk (talc). It is usual to put a little powder into the box with the finished pills; but if this is done the amount should be very small.

### Coatings for Pills.

Before leaving the subject of pills and passing on to consider the prescribing and dispensing of powders, it will be well to say something about the final processes which they often undergo after the making is complete, those, namely, of varnishing and coating with various materials.

Pills should never be coated with silver, talc, sugar, or anything of the kind, unless there is a good reason for it. The coating can but delay disintegration of the pill in the stomach of the patient. Sometimes, but seldom, such delay is indicated; there are, however, certain cases in which a coating of some kind is very desirable. Pills containing substances liable to be changed by contact with the air, either by oxidation or by absorption of moisture leading to swelling, deliquescence, or chemical change, and pills having an objectionable odour and taste, and pills containing a volatile ingredient, are all improved by varnishing.

(*To be continued.*)

